# Air restriction enhances *Streptococcus mutans* cariogenicity via preferential lactose metabolism

**DOI:** 10.1128/spectrum.03920-25

**Published:** 2026-04-29

**Authors:** Bowen Liu, Xian Li, Shasha Wang, Jingjing Xu, Wei Li, Huimin Yan, Min Li, Jingyi Yang

**Affiliations:** 1 Shanghai Public Health Clinical Center, Fudan University 12478, Shanghai, China; 2 Mucosal Immunity Research Group, State Key Laboratory of Virology, Wuhan Institute of Virology, Chinese Academy of Sciences, Wuhan, Hubei, China; University of Florida College of Dentistry, Gainesville, Florida, USA

**Keywords:** dental caries, *Streptococcus mutans*, cariogenicity, air restriction, sugar metabolism, lactose

## Abstract

*Streptococcus mutans* (*S. mutans*), a facultative anaerobe and lactic acid-producing bacterium, has been deemed a major etiological agent of dental caries. Our previous study revealed that *S. mutans* cultured under air-restricted conditions accelerated the development of dental caries in adult rats, compared with those grown in routinely air-unrestricted conditions. Here, we first confirmed this enhancement in a weaning rat model that mimics early childhood caries, and then further demonstrated that air restriction not only enhanced biofilm formation of *S. mutans,* but also upregulated gene expression in five Kyoto Encyclopedia of Genes and Genomes (KEGG) pathways associated with sugar uptake and metabolism, with lactose metabolism being most pronounced. Concurrently, genes involved in the conversion of pyruvate to acetyl-CoA were downregulated, shifting metabolic flux toward lactic acid production. This effect was markedly amplified in the presence of lactose, implying a critical role for lactose-derived fermentation in cariogenicity. Collectively, these findings indicate that air-restricted culturing enhances the cariogenic metabolic activity of *S. mutans*, with lactose emerging as a significant dietary risk factor for dental caries.

**IMPORTANCE:**

Dental caries remains one of the most prevalent chronic diseases worldwide, affecting billions of individuals globally, which poses a major burden to public oral health. A systematic understanding of environmental factors that modulate the virulence of cariogenic bacteria, such as *Streptococcus mutans* (*S. mutans*), provides valuable insights into the pathogenesis of caries and informs the development of prevention strategies. Our findings demonstrate that reduced air availability dramatically enhances the cariogenicity of *S. mutans in vitro* and *in vivo*, which is mechanistically associated with metabolic reprogramming toward preferential lactose utilization. Notably, our study bridges a critical knowledge gap by elucidating how environmental air tension drives *S. mutans* virulence and highlights lactose metabolism as a previously overlooked risk factor, prompting reconsideration of preventive strategies for vulnerable populations.

## INTRODUCTION

Dental caries, or tooth decay, is one of the most prevalent chronic diseases worldwide (1), affecting a majority of the global population (2, 3). Cariogenic biofilms, dominated by lactic acid-producing bacteria, metabolize dietary carbohydrates to generate lactic acid (4). This acid lowers the intraoral pH, leading to enamel demineralization. Prolonged demineralization ultimately results in dentin erosion and cavity formation (5). *Streptococcus mutans* (*S. mutans*), a Gram-positive facultative coccus, was identified as a cariogenic pathogen in the 1960s (6). As a primary colonizer during early childhood, *S. mutans* plays a central role in the initiation of dental caries, while other lactic acid bacteria, such as *Lactobacilli*, are more associated with the progression of caries (7). Although *S. mutans* has long been recognized as a primary causative agent of dental caries, current understanding places it within a broader ecological framework, where environmental changes and stresses disturb the balance of the microbial community, resulting in the development of a cariogenic microbiota (8, 9).

Decades of investigation on the cariogenicity of *S. mutans* (10) have delineated three key steps in dental caries development (11). First, *S. mutans* colonizes the tooth surface (12, 13). Second, the colonized *S. mutans* utilizes carbohydrates to synthesize exopolysaccharides, which form a matrix that coats the tooth surface and aggregates commensal bacteria, extracellular DNA, and proteins, thereby facilitating the formation of a cariogenic biofilm (14, 15). Finally, the *S. mutans* within the biofilm ferments carbohydrates to produce organic acids, primarily lactic acid, leading to tooth demineralization, dentin corrosion, and ultimately cavity formation (16). The resulting acidic microenvironment further promotes the proliferation of acid-tolerant oral flora while inhibiting acid-sensitive flora (15). Any factors influencing these three processes, including production of extracellular glucan polymers, carbohydrate transport and fermentation, acid tolerance, and stress adaptation, can modulate the cariogenic potential of *S. mutans* (17–19).

The oral cavity presents a complex ecosystem characterized by fluctuating nutrient availability, pH gradients, oxygen tensions, and competitive interactions between *S. mutans* and commensal oral microorganisms (20). *S. mutans* utilizes acid production, bacteriocin synthesis, and biofilm formation to dominate and promote a cariogenic environment. In contrast, commensal *streptococci* (e.g., *S. sanguinis*, *S. gordonii*) employ competitive strategies, including reactive oxygen species (ROS), to inhibit *S. mutans* overgrowth and maintain microbial balance (21). There are also synergy interactions between *S. mutans* and opportunistic pathogens, such as the fungal *Candida albicans. S. mutans* secreted glucosyltransferases (Gtfs), particularly GtfB, to bind *C. albicans* cell wall proteins, facilitating strong co-adhesion to enhance biofilm matrix production, promoting the formation of dense, mixed-species biofilms on tooth surfaces, and amplifying their pathogenic potential and adaptability (22). The co-presence of both pathogens is strongly associated with early childhood caries (ECC) severity (22).

As a facultative anaerobe, *S. mutans* cannot perform oxidative phosphorylation due to its incomplete electron transport chain (23). Oxygen molecules that enter *S. mutans* adventitiously obtain electrons, leading to the continuous generation of superoxide and hydrogen peroxide, which are collectively categorized as ROS (24). The accumulation of endogenous ROS elevates oxidative stress, potentially causing irreversible damage to metalloenzyme function and DNA integrity (25). To counteract ROS-mediated toxicity, *S. mutans* evolves multiple antioxidant enzymes, including superoxide dismutase (SOD) (24), alkyl hydroperoxide reductase (Ahp) (26), NADH oxidase (Nox) (27), and Dps-like peroxide resistance protein (Dpr) (28), which collectively maintain cellular homeostasis by scavenging intracellular ROS (29). Advances in the field have detailed the regulatory networks governing oxidative stress responses in *S. mutans* and their implications for bacterial survival in dynamic oral environments (29, 30). Within this context, *S. mutans* demonstrates remarkable metabolic flexibility, enabling it to utilize a variety of carbohydrates and rapidly adapt to environmental changes, a key determinant of its cariogenicity (31).

Comparative studies between aerobic and anaerobic conditions have revealed that oxygen availability affects the expression of biofilm-associated glucosyltransferases (32, 33), implying a potential role for oxygen in regulating cariogenicity of *S. mutans*. However, most prior studies have focused on contrasting strictly aerobic versus anaerobic conditions, leaving a gap in understanding how variations in oxygen tension, such as those occurring in the oral cavity during sleep, influence metabolic reprogramming and virulence. In our previous work, culturing *S. mutans* Ingbritt under air-restricted conditions reduced dissolved oxygen levels in the broth and upregulated the expression of virulence genes of *S. mutans*, compared with routinely air-unrestricted cultivation. We further demonstrated that *S. mutans* grown under air-restricted conditions exhibited markedly enhanced cariogenicity in adult rat models used for vaccine efficacy evaluation (34). Nevertheless, the mechanistic links between air restriction, metabolic rewiring, and enhanced caries formation remained unclear.

In this study, we showed that compared with air-unrestricted controls, air restriction-cultured *S. mutans* significantly exacerbates caries lesion severity in weaning rats, a well-established model in which caries development more closely resembles that in children. Notably, despite comparable viability of *S. mutans* under both culture conditions, dramatic phenotypic differences were observed. Transcriptomic profiling revealed that air restriction upregulated carbohydrate utilization pathways and enhanced lactic acid production, implicating these metabolic shifts as key factors of the heightened cariogenic potential of *S. mutans*.

## RESULTS

### Air-restricted culture promotes cariogenicity of *S. mutans* Ingbritt in weaning rats

In dental caries research, the weaning rat model is a well-established platform for evaluating the virulence of cariogenic bacteria and the efficacy of anti-caries vaccines and therapeutic interventions. Additionally, given that ECC has attracted more attention than caries in adults, we first investigated whether air-restricted culturing enhances the cariogenicity of *S. mutans* in weaning rats, as previously observed in adult rats (34). Specifically, for air-unrestricted culture, *S. mutans* Ingbritt was routinely grown in brain heart infusion (BHI) broth in conical flasks with air-permeable membranes. Air-restricted cultures were prepared in 15 mL screw-cap tubes filled with BHI broth.

**Fig 1:**
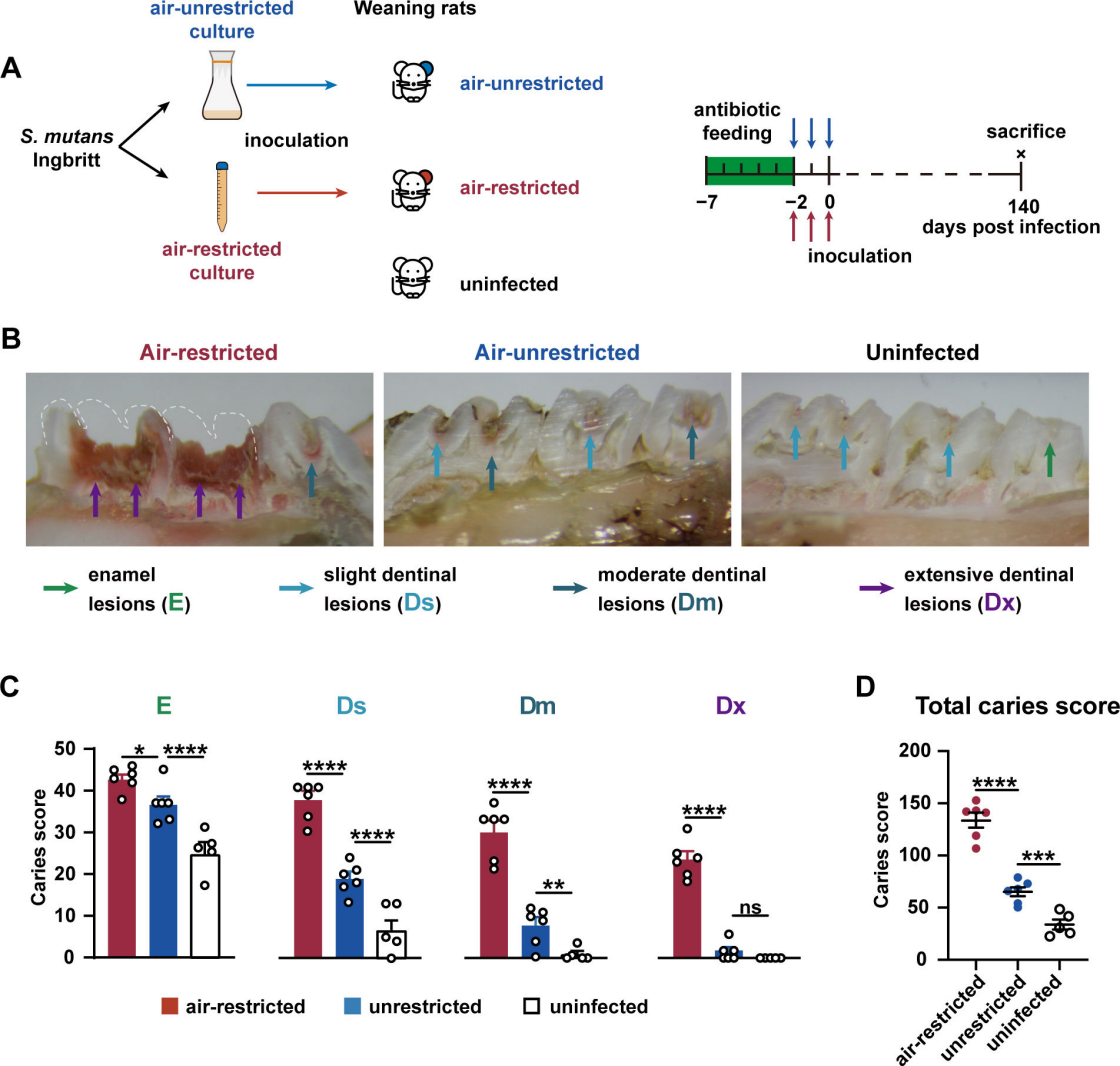
The effects of air-restricted culture on the cariogenicity of *S. mutans* Ingbritt in weaning rats. (**A**) Schedule of the *S. mutans* Ingbritt challenge experiment in weaning rats. Briefly, 18-day-old female rats were divided into three groups (five or six rats for each group). All rats in two challenge groups were fed a Keyes 2000 diet with multiple antibiotics for 5 consecutive days. Twelve hours before the challenge, the water and food containing antibiotics were removed and replaced with antibiotic-free Keyes 2000 diets. Then, the rats were orally infected with 1.7 × 10^9^ CFU of fresh *S. mutans* for 3 consecutive days (once a day). On day 140 post-infection, all rats, including uninfected rats, were sacrificed, and caries scores were determined. (**B**) Representative photographs of caries lesions in the indicated group on 140 days post-infection. The dashed line outlined the probable edge of the missing dental crown. (**C and D**) Caries scores of rats on day 140 post-infection. Total score = score of enamel lesions (**E**) + score of slight dentinal lesions (Ds) + score of moderate dentinal lesions (Dm) + score of extensive dentinal lesions (Dx). All data represent the mean ± SEM. Significance was determined by one-way ANOVA. *, *P* < 0.05; **, *P* < 0.01; ***, *P* < 0.001; ****, *P* < 0.0001; ns, nonsignificant.

Prior to inoculation with *S. mutans*, weaning rats were pre-treated with antibiotics to deplete native oral flora. The rats were then infected with *S. mutans* Ingbritt cultured under either air-unrestricted or air-restricted conditions for 3 days (Fig. 1A). A control group received no antibiotic treatment, bacterial challenge, or cariogenic diet. To allow the progression of dental caries to moderate stages, the experiment was extended to 140 days (35, 36). At 140 days post-inoculation (140 dpi), all rats were sacrificed to determine the caries lesions in the molar teeth (Fig. 1A). The results showed that extensive dentinal lesions (Dx) were rare in the air-unrestricted group but commonly observed in the air-restricted group (Fig. 1B and C). Notably, most rats in the air-restricted group exhibited crown loss in molars (Fig. 1B). Caries scores for enamel lesions (E), slight dentinal lesions (Ds), moderate dentinal lesions (Dm), Dx (Fig. 1C), and total lesions (Fig. 1D) were all significantly elevated following infection with *S. mutans* in the air-restricted group. These results demonstrate that air-restricted culture markedly enhances the cariogenic potential of *S. mutans* Ingbritt in weaning rats, compared with conventional air-unrestricted culture.

### *S. mutans* shows distinct phenotypes under air-restricted and air-unrestricted culture conditions

**Fig 2:**
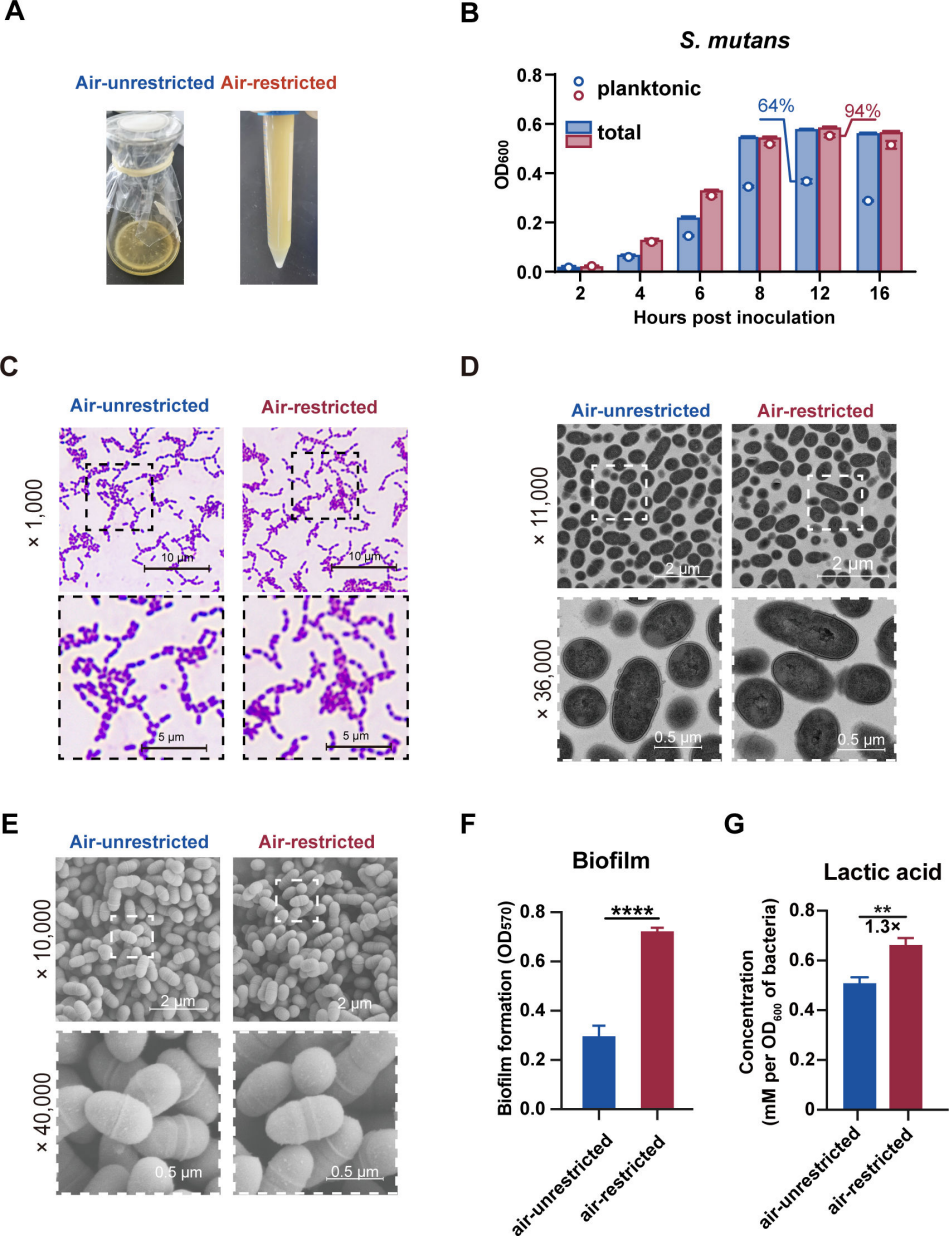
Phenotypic alterations of *S. mutans* Ingbritt under air-restricted conditions. *S. mutans* Ingbritt was added to a 150 mL conical flask containing 15 mL BHI broth with an air-permeable membrane or added to a 15 mL polystyrene centrifuge tube containing 15 mL BHI broth and statically cultured at 37°C. (**A**) The photographs of *S. mutans* under air-unrestricted conditions and air-restricted conditions for 12 h. (**B**) The OD_600_ values of the planktonic bacteria sampled from the middle of the broth without agitation and the thoroughly resuspended total bacteria. The planktonic bacteria OD values under the indicated condition is shown as a circle in each bar. The blue color represents the air-unrestricted group, and the red color represents the air-restricted group. (C–E) Morphology of *S. mutans* cultured statically under air-unrestricted conditions and air-restricted conditions for 12 h. Optical microscopic image (Gram stain) (**C**), TEM image (**D**), and SEM image (**E**) of Ingbritt. (**F and G**) biofilm formation ability (**F**) and lactic acid generation (**G**) of *S. mutans* Ingbritt cultured under air-restricted conditions or unrestricted conditions at 37°C for 12 h. All data represent the mean ± SEM. Significance was determined by Student’s *t*-test. **, *P* < 0.01; ****, *P* < 0.0001; ns, nonsignificant.

To explore mechanisms underlying the different cariogenicity of *S. mutans* cultured under air-restricted versus air-unrestricted conditions, we first characterized bacterial phenotypes. Consistent with previous reports indicating oxygen consumption during the growth of oral microbiota (37, 38), dissolved oxygen levels in BHI broth were extensively lower under air-restricted conditions compared with air-unrestricted conditions (Fig. S1). At 12 h post-inoculation (hpi), evident phenotypic disparities were observed between the two culture conditions. Precipitated bacteria were prominent in air-unrestricted cultures but minimal in air-restricted cultures (Fig. 2A).

Further analysis revealed that under air-unrestricted culture conditions, precipitated bacteria became apparent at 6 hpi during the logarithmic growth phase in broth (Fig. 2B, blue). By 12 hpi (stationary phase), substantial bacterial aggregation occurred, with only 64% of *S. mutans* Ingbritt remaining planktonic. In contrast, air-restricted cultures showed negligible precipitation during logarithmic growth (Fig. 2B, red), with 94% of bacteria remaining planktonic even at the stationary phase. Notably, no difference in cell viability was observed between the two conditions (Fig. S2). We hypothesized that morphological changes might explain these phenotypic differences. However, Gram staining (Fig. 2C), transmission electron microscope (TEM, Fig. 2D), scanning electron microscope (SEM, Fig. 2E) and revealed no apparent morphological differences in cell surface or inner structure of the bacteria between the air-restricted and air-unrestricted culture conditions.

Intriguingly, air-restricted *S. mutans* exhibited significantly enhanced biofilm formation capacity (Fig. 2F) and acid production (Fig. 2G) compared with air-unrestricted cultures. These findings suggest that the observed phenotypic variations likely stem from transcriptional and metabolic changes rather than morphological alterations.

### Air-restricted culture alters the transcriptional profile of *S. mutans* Ingbritt

**Fig 3:**
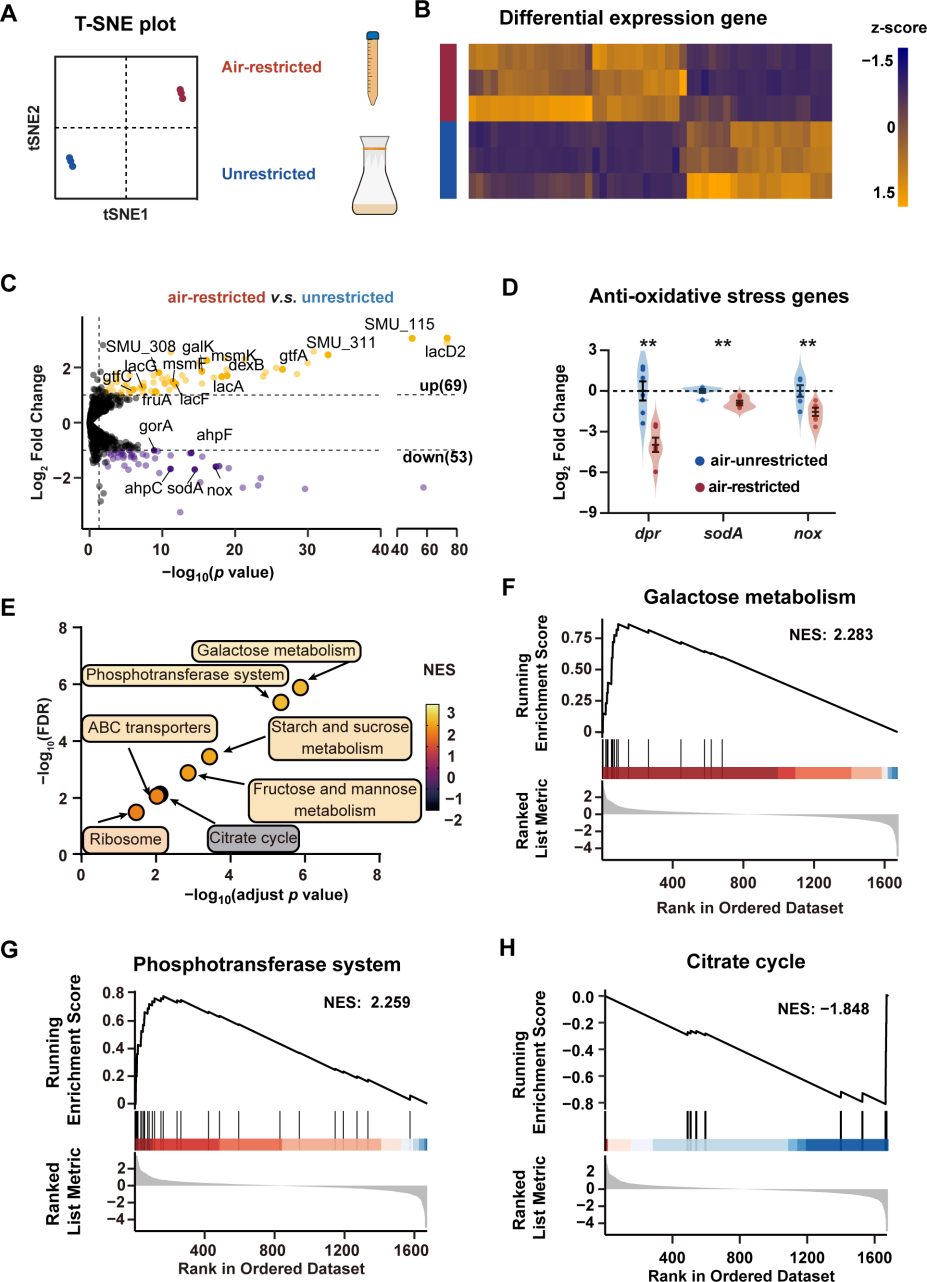
The effect of air-restricted culture on the transcriptional profile of *S. mutans*. (**A**) t-SNE analysis of the gene expression profile (*n* = 1,913) by RNA-seq analysis and (**B**) heatmap of the top 50 DEGs between the air-restricted cultured *S. mutans* Ingbritt and unrestricted cultured *S. mutans* Ingbritt. (**C**) Volcano plot of all DEGs filtered by |log_2_-fold change| > 1 and *P* value <0.05. The downregulated genes, nonsignificantly changed genes, and upregulated genes are shown in purple, gray, and yellow, respectively (air-restricted versus unrestricted). The *P* value was determined by using DESeq2’s likelihood ratio test (LRT) in the R language (version 4.2.0). (**D**) RT-qPCR was used to verify the expression level changes in anti-oxidative stress genes. (**E**) The bubble plot shows significantly changed Kyoto Encyclopedia of Genes and Genomes (KEGG) pathways according to gene set enrichment analysis (GSEA). The adjusted *P* value was determined by the permutation test, and the FDR was determined by multiple-hypothesis testing using the Benjamini method. NES, normalized enrichment score. (**F–H**) GSEA plot depicting the gene enrichment changes in the galactose metabolism pathway (**F**), phosphotransferase system pathway (**G**), and citrate cycle (**H**). Significance was determined by Student’s *t*-test.**P < 0.01.

To elucidate the rational mechanism underlying the observed phenotypic changes, we performed parallel transcriptomic analysis of *S. mutans* Ingbritt cultured under air-restricted and air-unrestricted conditions. Both t-distributed stochastic neighboring embedding (t-SNE) analysis (Fig. 3A) and heatmap visualization of the top 50 differentially expressed genes (DEGs) (Fig. 3B) revealed substantial transcriptional alterations under air-restricted conditions. Comparative analysis identified 122 significant DEGs (|log_2_-fold change| > 1 and *P* value <0.05) comprising 69 upregulated and 53 downregulated genes (Fig. 3C; Table S1). Consistent with the reduced oxygen availability (Fig. S1), we observed downregulation of genes encoding oxidative stress defense enzymes (39) (Fig. 3C, dark purple). Selected DEGs were validated by real-time reverse transcription PCR (RT-qPCR) (Fig. 3D).

Gene set enrichment analysis (GSEA) identified seven significantly altered Kyoto Encyclopedia of Genes and Genomes (KEGG) pathways (adjusted *P* value <0.05, FDR < 0.25, and |NES| > 1.5) (Fig. 3E; Table S2). Notably, six out of seven pathways were upregulated (NES > 1.5) under air-restricted conditions, including five KEGG pathways associated with carbohydrate uptake and metabolism (galactose metabolism, phosphotransferase system [PTS], fructose/mannose metabolism, starch/sucrose metabolism, and ATP-binding cassette [ABC] transporters) (Fig. 3F and G; Fig. S3). The other upregulated KEGG pathway was the ribosome pathway, which involved genes encoding ribosome proteins. On the other hand, the only significantly downregulated KEGG pathway was the citrate cycle pathway (NES < −1.5) (Fig. 3H).

### Air-restricted culture enhances carbohydrate transportation and metabolism with preferential galactose utilization

**Fig 4:**
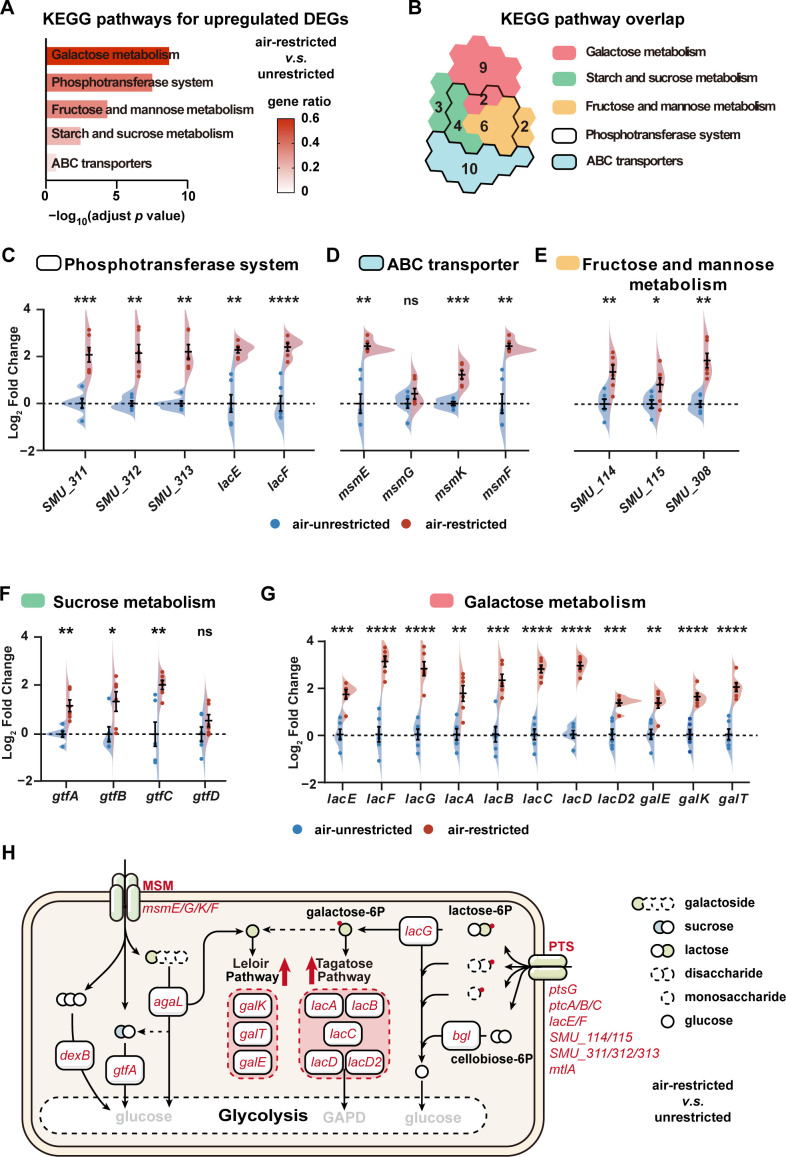
The KEGG enrichment analysis of differentially expressed genes. (**A**) The column plot shows the upregulated DEG-enriched KEGG pathways. The filled color represents the ratio of upregulated genes in the pathway. Significance was determined by hypergeometric distribution analysis, and the *P* values were calculated by Fisher’s exact test. (**B**) The number of upregulated DEGs in the five indicated KEGG pathways. (**C–G**) RT-qPCR was used to assay the expression level changes in genes enriched in phosphotransferase system pathway (**C**), ABC transporter pathway (**D**), fructose and mannose metabolism pathway (**E**), starch and sucrose metabolism pathway (**F**), and galactose metabolism pathway (**G**). Relative expression of the genes was normalized by double reference genes (*16S rRNA* and *gyrA*). (**H**) Schematic summary of upregulated genes associated with carbohydrate uptake and utilization. PTS, phosphotransferase system; MSM, multiple sugar metabolism system. Upregulated genes in the air-restricted group (compared to the air-unrestricted group) identified by transcriptomics were colored in red. Significance was determined by Student’s *t*-test. *, *P* < 0.05; **, *P* < 0.01; ***, *P* < 0.001; ****, *P* < 0.0001; ns, nonsignificant.

Further analysis of DEGs (40) (adjust *P* value <0.05) revealed that the 69 upregulated DEGs under air-restricted conditions were significantly enriched in five upregulated KEGG pathways (Fig. 4A; Table S3), which precisely matched the top five upregulated KEGG pathways by GSEA (Fig. 3E). Notably, both analysis identified galactose metabolism as the most significantly enriched pathway, indicating its potential role as a dominant carbohydrate utilization route in *S. mutans* grown without sufficient air.

Additionally, all upregulated DEGs in the PTS pathway and four upregulated DEGs in the ABC transporter pathway were specifically involved in sugar transportation (Table S3). The PTS pathway DEGs showed complete overlap with those in the galactose, fructose/mannose, and starch/sucrose metabolism pathways (Fig. 4B). Supporting these findings, Gene Ontology (GO) analysis confirmed enrichment of transportation-related functions among upregulated DEGs (Fig. S4).

RT-qPCR validation subsequently confirmed most of the transcriptomic findings (Fig. 4C through G). Both biofilm-associated glucosyltransferase genes (*gtfB*/*gtfC*) and the intracellular sucrose hydrolase genes (*gtfA*) in the sucrose metabolism pathway were significantly upregulated under air-restricted conditions (Fig. 4F). In the most prominently upregulated galactose metabolism pathway, all 11 enzyme-encoding genes were coordinately upregulated (Fig. 4G).

In the galactose metabolism pathway and the sucrose metabolism pathway, we found that genes encoding permeases that transport oligosaccharides, including sucrose (*msmE*/*F*/*G*/*K*) (41, 42) and lactose (*lacE*/*lacF*) (43), were both upregulated (Fig. 4D, E, and H). For the starch and sucrose metabolism pathway, five upregulated DEGs were associated with the internalization and hydrolyzation of cellobiose (*ptcA/B/C* and *bgl*), and transportation of maltose (*ptsG*), instead of those related to sucrose (Fig. 4H; Table S3). The coordinated upregulation of 12 genes in the PTS pathway and 4 genes in the ABC transporters pathway strongly indicated enhanced sugar internalization under air-restricted culture conditions.

Following internalization, oligosaccharides are subsequently hydrolyzed to monosaccharides. Our analysis showed that genes encoding the sucrose hydrolase (*gtfA*) and 6-phospho-β-galactosidase (*lacG*) that specifically hydrolyze sucrose and lactose-6-phosphate, respectively, were upregulated under air-restricted culture conditions (Fig. 4H). Besides these, two additional genes encoding galactoside hydrolase (*agaL*, α-galactosidase) and dextran hydrolase (*dexB*, intracellular glucan 1, 6 α-glucosidase) were also significantly upregulated (Fig. 4H; Table S1), suggesting an increase in sugar catabolism of *S. mutans* grown under air-limited conditions.

Notably, in the metabolism of galactose, both the Tagatose and Leloir pathways showed universal gene upregulation, ensuring efficient conversion of galactose-6-phosphate (Gal-6-P) and galactose into glycolytic intermediates (43) (Fig. 4H). These coordinated changes indicate that air-restricted conditions enhance metabolic flux through galactose utilization pathways in *S. mutans*.

### Air-restricted culture enhances lactic acid production preferentially stimulated by lactose

**Fig 5:**
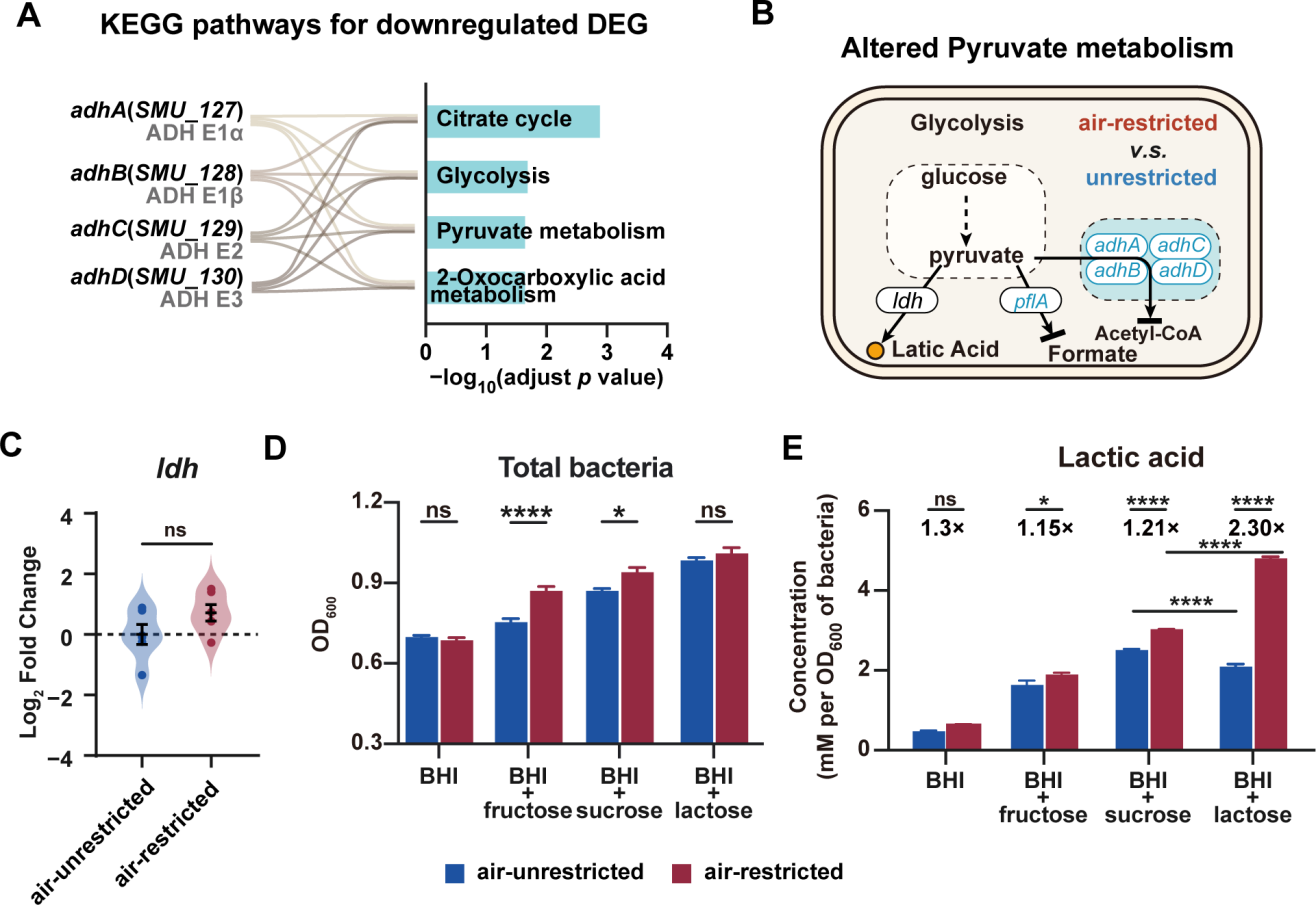
Effects of air-restricted culture on the metabolism of sugar. (**A**) The downregulated DEGs enriched in pathways. The column plot shows the downregulated DEG-enriched KEGG pathways. The connecting lines indicate genes encoding the ADH complex can be enriched in each KEGG pathway. Significance was determined by hypergeometric distribution analysis, and the adjusted *P* values were calculated by Fisher’s exact test. (**B**) The schematic of altered pyruvate metabolism in *S. mutans* under air-restricted conditions compared to that under air-unrestricted conditions. (**C**) The expression level of lactic dehydrogenase in *S. mutans* after 12 h of culture. (**D and E**) *S. mutans* were cultured in BHI broth with or without an extra 10 mM sugar (fructose, sucrose, or lactose) statically at 37°C for 12 h. (**D**) The OD_600_ value of *S. mutans* under air-restricted and unrestricted conditions with or without extra sugars. (**E**) The level of lactic acid production in *S. mutans*. The bacterial pellets were collected by centrifugation, washed with ddH_2_O, and lysed with a Beater homogenizer. The intracellular lactic acid was extracted into ddH_2_O. The measured data represent the mean ± SEM. For panel **C**, significance was determined by Student’s *t*-test. For panels D and **E**, significance was determined by two-way ANOVA. *, *P* < 0.05; ***, *P* < 0.001; ****, *P* < 0.0001; ns, nonsignificant.

Analysis of the 53 downregulated DEGs identified four significantly enriched KEGG pathways (adjusted *P* value <0.05), all of which contain the same set of genes (*adhA*, *adhB*, *adhC*, and *adhD*) encoding acetoin dehydrogenase complex (ADH) subunits that convert pyruvate into acetyl-CoA (Fig. 5A and B). Additionally, pyruvate formate-lyase (*pflA*), which converts pyruvate to formate, was also downregulated under air-restricted culture conditions (Table S1). Importantly, lactate dehydrogenase (*ldh*) expression remained unchanged (Fig. 5C), indicating that the air-restricted conditions specifically reduce the pyruvate flux toward acetyl-CoA while maintaining the pyruvate flux toward lactic acid (Fig. 5B). Combined with our findings of enhanced glycolytic input under air-restricted culture conditions (Fig. 4), these results suggest a trend of increased lactic acid production under air-restricted conditions. Consistent with the hypothesis, we observed a slightly elevated lactic acid level in BHI after culturing for 12 h under air-restricted conditions (Fig. 5E).

To evaluate sugar-specific effects on lactic acid production, we supplemented BHI broth with 10 mM fructose, lactose, or sucrose. All sugars stimulated bacterial proliferation and lactic acid generation under both culture conditions (Fig. 5D and E). Notably, lactose elicited the strongest response in air-restricted cultures, increasing intracellular lactic acid by 1.3-fold, whereas sucrose or fructose resulted in less than 25% increase (Fig. 5E). Similarly, extracellular lactic acid levels also significantly increased upon addition of extra sugars, especially under air-restricted conditions, although to a lesser extent (Fig. S5). These results demonstrate that air-restricted conditions preferentially enhance lactose-driven lactic acid production and bacterial proliferation relative to other tested sugars.

## DISCUSSION

As a facultative anaerobic bacterium, ROS was harmful for the growth of *S. mutans*, while *S. mutans* evolved several anti-oxidative stress enzymes to scavenge limited ROS (39). In the oral cavity, oxygen levels may decrease during periods of restricted air supply (e.g., sleep) due to microbial growth (37, 38, 44). However, how oxygen supply limitations affect the cariogenicity of *S. mutans* remains unclear.

Our previous study demonstrated that the air-restricted cultured *S. mutans* showed higher cariogenicity in adult rats (34), suggesting oxygen might be an important factor in altering the cariogenicity of *S. mutans*. This study extended these findings to weaning rats (18-day-old), showing that *S. mutans* cultured under air-restricted conditions caused significantly more severe caries, compared with that under air-unrestricted conditions. At 140 dpi, weaning rats challenged by air-restricted *S. mutans* exhibited higher caries score and molar crown loss, whereas those challenged by air-unrestricted *S. mutans* showed only slight or moderate lesions (Fig. 1), confirming that air restriction enhances cariogenicity regardless of rat age.

Previous studies have established that oxygen availability influences key virulence traits in *S. mutans*, such as biofilm formation and glucosyltransferase expression (32, 33). Our study further demonstrated that air restriction, resembling conditions that may occur in the oral cavity when airflow is reduced, not only promotes a planktonic state but also enhances biofilm capacity associated with increased cariogenicity (Fig. 2). This suggests that oxygen availability may serve as an environmental signal that influences how *S. mutans* balances dispersal and colonization strategies. Under low air supply, the bacteria tend to remain suspended to explore broader territories, while staying ready to form robust biofilms when conditions become favorable. This adaptive mechanism may help *S. mutans* compete and survive in the complex oral microenvironment.

Transcriptional data analysis revealed that air-restricted conditions upregulated adhesion-associated genes (Fig. S6) and biofilm-associated glucosyltransferase genes (Fig. 4) of *S. mutans*. This observation is generally consistent with earlier transcriptional comparisons of *S. mutans* cultured under anaerobic and aerobic conditions (32), although certain genes we found to be significantly upregulated, such as *gtfC* and *gbp,* were not markedly altered in the previous study. These differences might be attributed to the use of distinct *S. mutans* strains and different culturing systems adopted. Additionally, we found that air restriction downregulated oxidative stress defense genes (e.g., *sod*, *ahp*, *nox*) (Fig. 3C and D), consistent with reduced ROS generation under low oxygen. Meanwhile, we observed a marked upregulation of carbohydrate transport and metabolism pathways, particularly those for galactose/lactose utilization (Fig. 3 and 4). This reciprocal regulation suggests that low oxygen may prompt *S. mutans* to shift resources from oxidative stress defense to enhanced sugar catabolism, a strategy that maximizes energy yield and acid production in oxygen-limited conditions.

A key finding of our study is the upregulation of acidogenic metabolic pathways under air-restricted conditions, particularly lactose metabolism (Fig. 3 to 5). Key upregulated pathways included sugar transport systems (MSM and PTS) and metabolic processes, with the galactose pathway showing particularly strong activation (Fig. 3 and 4). The lactose metabolic pathway deserves special attention. In *S. mutans*, lactose is transported into the cell via lactose-specific PTS transporter (*lacE* and *lacF*) and phosphorylated, cleaved by 6-phosphate-β-galactosidase (*lacG*) into glucose and galactose-6-phosphate. Our data show coordinated upregulation of all genes in both the tagatose and Leloir pathways under air restriction (Fig. 4H), indicating preferential galactose utilization.

As both the terminal product of glycolysis and a pivotal metabolic node, pyruvate links energy production, biosynthesis, and stress adaptation (23). In *S. mutans*, the metabolic fate of pyruvate directly dictates core virulence traits, including acid production, biofilm formation, and oxidative stress tolerance (45). Our data indicated that air restriction downregulated pyruvate conversion to acetyl-CoA and formate while maintaining lactate dehydrogenase (*ldh*) expression (Fig. 5), effectively channeling more pyruvate toward lactic acid production. This metabolic redirection, combined with enhanced sugar uptake, explains the observed increase in acid production, particularly with lactose supplementation (Fig. 5).

Our data further revealed that, under air-restricted conditions, lactose supplementation led to a significantly higher intracellular lactic acid level than sucrose supplementation (Fig. 5E). However, the increase in extracellular lactic acid induced by lactose did not differ significantly from that induced by sucrose (Fig. S5). It is important to note that extracellular lactic acid levels reflect the accumulation of lactic acid resulting from acid production, efflux, degradation, and neutralization by medium components. Moreover, extracellular lactic acid levels under air-restricted conditions may also be affected by lactic acid generation during the initial phase of culture (e.g., the first 4 h). During this period before oxygen consumption, which resembled air-unrestricted conditions, sucrose stimulated higher acid production than lactose (Fig. 5). These combined factors may explain the lack of a significant difference between lactose and sucrose in extracellular lactic acid accumulation. Nonetheless, the observed increasing trend still supports the conclusion of enhanced lactic acid production under air-restricted conditions. It is worth noting that this experiment has a limitation that warrants consideration: the amounts of lactose and sucrose (disaccharides, MW = 342) are not equivalent to fructose (monosaccharide, MW = 180) on the same mole basis, and the metabolism of fructose into lactic acid might be slightly underestimated.

The observed transcriptional changes, while not directly upregulating classic acid tolerance-related genes, such as those encoding F_1_F_0_-ATPase, nonetheless suggest a mechanism by which air restriction could enhance functional aciduricity. Upregulation of sugar transport and glycolytic pathways can also increase ATP generation, thereby fueling proton extrusion systems essential for intracellular pH homeostasis (23, 27). Moreover, the enhanced lactic acid production itself could contribute to acid adaptation by selecting for acid-tolerant phenotypes within the population or by promoting the formation of a protective acidic biofilm matrix (15, 16). Our data revealed upregulation of adhesion-associated genes (*spaP/gbpB*) (Fig. S6) and biofilm-associated glucosyltransferase genes (*gtfB/gtfC*) (Fig. 4), suggesting that air restriction may promote acid tolerance by enhancing biofilm formation and surface adherence. Collectively, air restriction may prime *S. mutan*s not only to produce more acid but also to better withstand the low-pH environment it creates, a dual advantage in caries pathogenesis.

In the oral ecosystem, *S. mutans* competes with numerous other microorganisms. As a pioneer colonizer, *S. mutans* actively modifies its environment by consuming oxygen and producing acid, creating favorable conditions for itself and other acid-tolerant species like *Lactobacillus* (15). Our transcriptomic data showed upregulation of genes related to bacteriocin resistance (*SMU_1006/1007*) and hemolysin-like transporters (*SMU_431/432*) (Table S3), although classic mutacin biosynthesis genes were not induced. These findings suggest that under air restriction, *S. mutans* may utilize a defensive or tolerance strategy rather than an offensive approach involving bacteriocin production. This ecological advantage, combined with increased acid production, likely explains the observed cariogenicity enhancement. While our omics data reveal clear connections between air-restriction stress and carbohydrate metabolism, the precise regulatory mechanisms require further investigation.

Clinically, our findings highlight lactose as a previously underappreciated dietary risk factor for caries, especially in the context of reduced oral oxygen tension during sleep (37, 38, 44). Infants and young children who consume milk before bedtime may therefore be at higher risk, given that low-oxygen conditions prime *S. mutans* to ferment residual lactose more efficiently. This insight emphasizes the importance of oral hygiene routines and dietary habits in early childhood caries prevention.

In summary, air restriction enhances *S. mutans* cariogenicity by promoting biofilm formation and lactose-driven acidogenesis, identifying lactose as a key dietary risk factor during sleep-related hypoxia, warranting special preventive measures for children’s nighttime milk intake.

## MATERIALS AND METHODS

### Bacterial strains

The *S. mutans* strain Ingbritt was stored in our laboratory and propagated in BHI broth (Oxoid).

### Bacteria culture and measurements

*S. mutans* was cultured statically in BHI broth (Oxoid) at 37°C. For air-restricted conditions, bacteria were grown in 15 mL screw-cap polypropylene tubes filled completely with BHI broth, with caps tightened to minimize gas exchange. For air-unrestricted cultures, 15 mL BHI broth was dispensed into 150 mL sterile conical flasks sealed with an air-permeable vented membrane (hydrophobic PTFE membrane, 0.2–0.3 μm pore size), allowing continuous air exchange while preventing contamination. All cultures were incubated in a standard aerobic incubator with ambient air at 37°C. Planktonic bacteria were sampled by collecting 200 μL from the mid-broth without agitation. Total bacteria were measured after complete resuspension. OD_600_ was determined using a BioTek spectrophotometer. Dissolved oxygen levels were recorded in undisturbed broth using an INESA oxygen meter.

### Rat experiments

The *S. mutans* Ingbritt infection procedure was performed as previously described (36). Specific pathogen-free 18-day-old female Wistar rats from Wuhan Institute of Biological Products were housed in individually ventilated cages at Wuhan Institute of Virology’s Animal Center.

Seventeen rats were randomly divided into uninfected (*n* = 5) and two challenge groups (*n* = 6 each). After a 3-day acclimation, rats received Keyes 2000 cariogenic diet (56% sucrose, 28% milk powder) and antibiotic water (1 g/L ampicillin/chloramphenicol/carbenicillin) for 5 days. Twelve hours pre-infection, antibiotics were discontinued. *S. mutans* Ingbritt cultured under air-restricted or unrestricted conditions for 12 h was resuspended in BHI broth (3.4 × 10^9^ CFU/mL). Rats received 1.7 × 10^9^ CFUs daily for 3 consecutive days (day 0 was the final infection). Caries scores were evaluated at day 140 post-infection. Teeth were collected, cleaned, and stained with 0.4% murexide in 70% ethanol according to established protocols (36, 46, 47); then were washed, hemisectioned, and examined under a stereomicroscope (Phenix Optics) for caries scoring using the Keyes method (48).

### Animal welfare monitoring and humane endpoints

Throughout the 140-day experimental period, all animals were monitored weekly for general health and welfare by trained personnel. Monitoring included assessment of activity level, posture, coat condition, respiration, and signs of pain or distress (e.g., facial rubbing, reduced grooming, or abnormal vocalization). Body weight was recorded weekly as an objective indicator of nutritional status and overall health. Predefined humane endpoints were established prior to the study and strictly adhered to, including (i) weight loss exceeding 20% of baseline body weight; (ii) severe lethargy, dyspnea, or inability to access food or water; (iii) clinical signs of irreversible pain or distress. No animal met any of these criteria during the study; therefore, all rats completed the planned experimental duration.

### Microscopy analysis

*S. mutans* cultures (12-h growth) were centrifuged and washed six times with NaCl-free PBS. Bacterial pellets (5–10 mg wet weight) were fixed in 2.5% glutaraldehyde, rinsed with 0.1 M phosphate buffer (NaCl-free), and dehydrated through an ethanol series. For SEM, samples underwent critical point drying, were mounted on stubs, and sputter-coated with ~10 nm gold prior to imaging at 5–15 kV. For TEM analysis, the washed bacterial samples underwent post-fixation with 1% osmium tetroxide, followed by dehydration, stepwise epoxy resin infiltration using progressively increasing resin to acetone ratios, and subsequent pure resin polymerization. Ultrathin sections were double-stained with 2% uranyl acetate and lead citrate before TEM analysis. All electron microscopy procedures were conducted at the Center for Instrumental Analysis and Metrology, Wuhan Institute of Virology, CAS.

### Biofilm assay

*S. mutans* Ingbritt was first cultured under air-restricted or unrestricted conditions in BHI broth for 12 h at 37°C, and then diluted 1:100 in Tryptic Soy Broth containing 1% sucrose. Aliquots (100 μL) were transferred to 96-well plates and incubated anaerobically at 37°C for 10 h to allow biofilm formation to occur. After washing with ddH₂O, biofilms were stained with crystal violet (Beyotime Biotechnology) for 10 min, washed again, and solubilized with 30% acetic acid. Absorbance was measured at 570 nm using a BioTek spectrophotometer.

### Lactic acid assay

Lactic acid quantification was performed using a commercial assay kit (Nanjing Jiancheng Bioengineering Institute). *S. mutans* Ingbritt cultures grown for 12 h at 37°C under air-restricted or unrestricted conditions, with or without 10 mM sugar supplementation, were centrifuged (12,000 × *g*, 4°C, 1 min) and washed with cold ddH_2_O. Bacterial pellets were homogenized with 0.1 mm glass beads in a 4°C bead beater (4 × 30 s cycles with 1 min intervals) and resuspended in ddH2O prior to lactic acid measurement according to the manufacturer’s protocol.

### RNA-Seq and analysis

*S. mutans* cultures grown under air-restricted and unrestricted conditions for 12 h were centrifuged and flash-frozen in liquid nitrogen. Wuhan Bioacme Company conducted mRNA extraction, sequencing, and initial bioinformatics analysis. Total RNA was extracted, rRNA-depleted, and mRNA-enriched for cDNA library preparation prior to HiSeqTM2000 sequencing. FASTQ file quality was assessed using Fastp (v0.20.0), with quality-filtered reads aligned via HISAT2 against the UA159 strain reference genome (GCF_000007465.2), as no Ingbritt strain assembly was available in NCBI. Downstream analyses employed R (v4.2.0) with the Rtsne package for t-SNE (49) visualization. DESeq2 (v1.34.0) identified differentially expressed genes, while clusterProfiler (v4.2.0) (40) performed KEGG, GO, and GSEA enrichment analyses.

### RNA extraction and quantitative reverse transcription polymerase chain reaction

Following 12-h culture at 37°C, *S. mutans* cells were pelleted by centrifugation (12,000 × *g*, 4°C, 1 min), washed twice with cold DEPC-treated water, and mechanically lysed using 0.1 mm glass beads in a 4°C bead beater (4 × 30 s cycles with 1 min intervals). Total RNA was extracted using the FastPure RNA Isolation Kit (Vazyme), with quality verified by gel electrophoresis and Nanodrop 2000 spectrophotometry (A260/A280 ≥ 2.0). cDNA synthesis and qPCR amplification were performed using HiScript II One Step SYBR Green Kit (Vazyme) on a Bio-Rad system, with expression levels normalized to *16SrRNA* and *gyrA* reference genes (50). Primer sequences are listed in Table S4.

### Statistics

Statistical analyses were performed using GraphPad Prism 9.4 (excluding transcriptomic data). Normally distributed data with equal variance were analyzed by ANOVA or unpaired two-tailed *t*-test, while nonparametric data were analyzed using the Mann-Whitney *U* test. Significance levels were denoted as *, *P* < 0.05; **, *P* < 0.01; ***, *P* < 0.001; ****, *P* < 0.0001; ns, not significant (α = 0.05).

## Data Availability

The raw sequencing data reported in this paper have been deposited in the Genome Sequence Archive (51) in the National Genomics Data Center, that are publicly accessible at https://ngdc.cncb.ac.cn/gsa (GSA: CRA028242).

## SUPPLEMENTAL MATERIAL

Supplemental material spectrum.03920-25-s0001.pdf Fig. S1 to S6; Tables S1 to S4.

ASM does not own the copyrights to Supplemental Material that may be linked to, or accessed through, an article. The authors have granted ASM a non-exclusive, world-wide license to publish the Supplemental Material files. Please contact the corresponding author directly for reuse.
