# Supplementary material for: Air restriction enhances Streptococcus mutans cariogenicity via preferential lactose metabolism
Source: Microbiol Spectr. 2026 Apr 29;14(6):e03920-25. doi: 10.1128/spectrum.03920-25 (PMC13228075; doi:10.1128/spectrum.03920-25)
Supplement: Supplemental material — Fig. S1 to S6; Tables S1 to S4. [file spectrum.03920-25-s0001.pdf]

Supplemental Material

Supplemental Figures

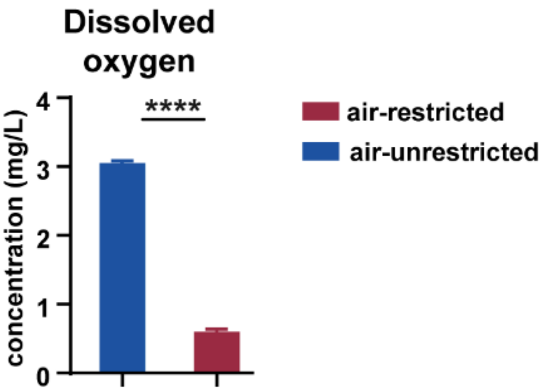

Figure S1. The dissolved oxygen level in broth at 12 hours post-inoculation. *S. mutans* Ingbritt was added to a 150 mL conical flask containing 15 mL BHI broth with an air-permeable membrane or added to a 15 mL polystyrene centrifuge tube containing 15 mL BHI broth and statically cultured at 37°C for 12 hours. A dissolved oxygen meter was inserted into the middle of the broth without agitation to assess the dissolved oxygen concentration. Data represent the mean ± SEM. Significance was determined by Student's t-test. ****, $p < 0.0001$.

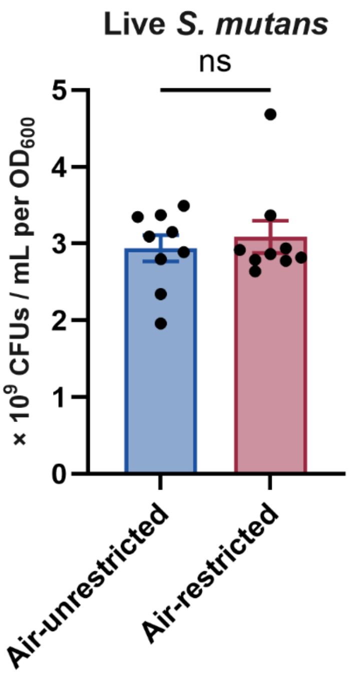

Figure S2. The ratio of live *S. mutans* density (CFU/mL) to OD_{600} at 12 hours post-culture. *S. mutans* Ingbritt was added to a 150 mL conical flask containing 15 mL BHI broth with an air-permeable membrane or added to a 15 mL polystyrene centrifuge tube containing 15 mL BHI broth and statically cultured at 37°C. *S. mutans* was cultured under the indicated conditions and quantified colony-forming units of live total bacteria on MSB plates. Data represent the mean ± SEM. ns, nonsignificant.

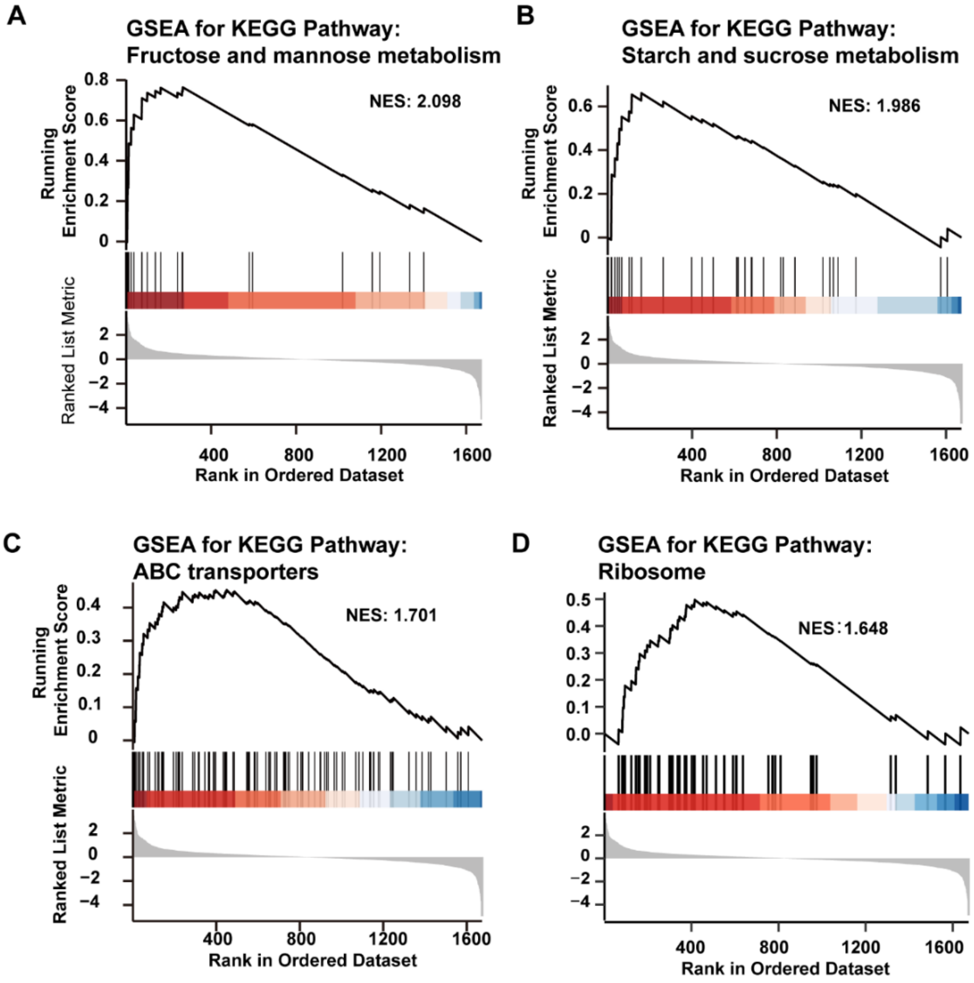

Figure S3. GSEA analysis of enriched KEGG pathway changes based on all tested genes. GSEA plot depicting the gene enrichment changes in KEGG pathways of *S. mutans* Ingbritt cultured under air-restricted conditions versus air-unrestricted conditions (fructose and mannose metabolism pathway, starch and sucrose metabolism pathway, ABC transporter pathway, and ribosome pathway). NES, normalized enrichment score; FDR was corrected by the Benjamini-Hochberg method.

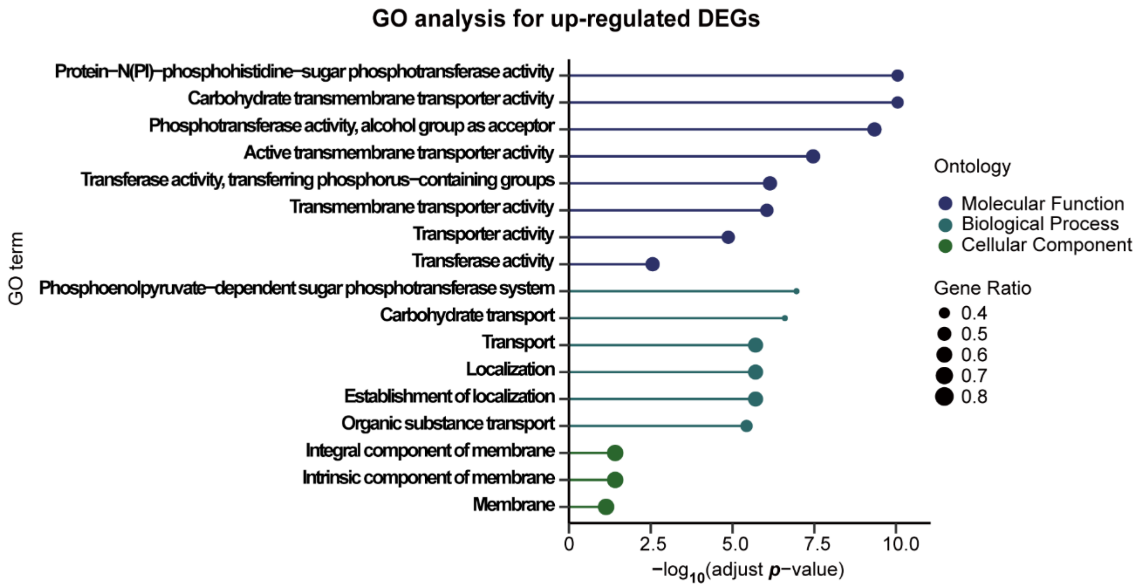

Figure S4. GO analysis of the upregulated DEGs of *S. mutans* Ingbritt cultured under air-restricted conditions compared to that under air-unrestricted conditions. BP, CC, and MF subentries of GO analysis based on upregulated DEGs by using the cluster Profiler package. Significance was determined by hypergeometric distribution analysis, and the *p* values were calculated by Fisher’s exact test.

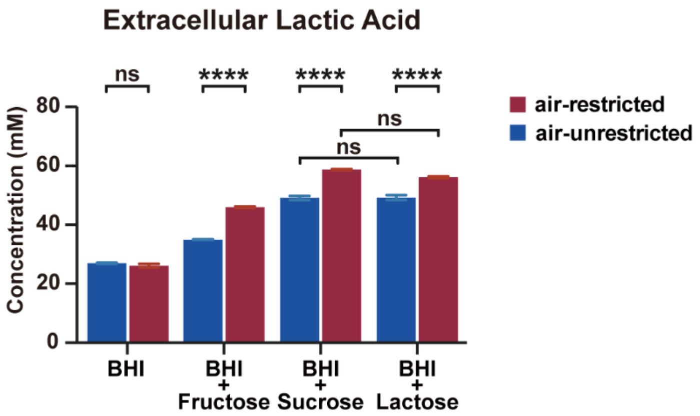

Figure S5. The level of extracellular lactic acid (Related to Fig.5). After 12-hour culturing of *S. mutans* in BHI with or without sugar supplement, extracellular lactic acid in media was assayed. The measured data represent the mean ± SEM. Significance was determined by two-way ANOVA. ****, $p < 0.0001$; ns, nonsignificant.

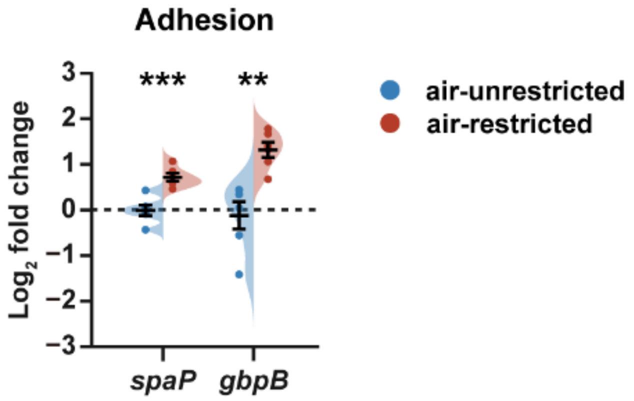

Figure S6. The adhesion-related gene expression of *S. mutans* Ingbritt under two air-restriction conditions. *S. mutans* was cultured statically under air-restricted conditions or air-unrestricted conditions. The transcriptional level of genes associated with *S. mutans* adhesion was determined by qRT–PCR. The relative expression was normalized to double reference genes (*16SrRNA* and *gyrA*). All data are represented as mean ± SEM. Statistical significance was determined by unpaired two-tailed Student's t-test. **, $p < 0.01$; ***, $p < 0.001$.

Supplemental Tables

Table S1. Genes that are differentially expressed between the air-restricted group and the air-unrestricted group.

Gene ID	Genes	$\log_2$(Fold Change) (air-restricted vs. unrestricted)	$-\log_{10}(p\text{-value})$
SMU_116	*lacD2*	3.055	73.97
SMU_115	*NA*	3.045	50.12
SMU_114	*NA*	2.879	73.88
SMU_1881c	*NA*	2.81	1.85
SMU_113	*pfkB*	2.577	30.96
SMU_1597c	*NA*	2.548	11.15
SMU_311	*NA*	2.458	32.92
SMU_312	*NA*	2.327	18.62
SMU_148	*adhE*	2.299	21.25
SMU_882	*msmK*	2.256	16.06
SMU_431	*NA*	2.157	30.14
SMU_310	*NA*	2.154	14.95
SMU_1596	*ptcC*	1.983	25.7
SMU_881	*gtfA*	1.933	26.62
SMU_1601	*bgl*	1.921	18.85
SMU_886	*galK*	1.864	15.41
SMU_21	*mreD*	1.844	1.51
SMU_309	*NA*	1.819	24.38
SMU_308	*NA*	1.814	9.53
SMU_654	*mutF*	1.778	3.56
SMU_1599	*celR*	1.761	28.77
SMU_887	*galT*	1.712	12.7
SMU_868	*trmD*	1.708	5.5
SMU_1595	*cah*	1.705	8.8
SMU_883	*dexB*	1.702	18.95
SMU_880	*msmG*	1.67	23.99
SMU_1496	*lacA*	1.667	18.23
SMU_432	*NA*	1.631	17.14
SMU_1598	*ptcA*	1.63	10.7

Gene ID	Genes	$\log_2$(Fold Change) (air-restricted vs. unrestricted)	$-\log_{10}$(p-value)
SMU_1550c	*NA*	1.598	3.85
SMU_313	*NA*	1.587	9.96
SMU_1957	*NA*	1.534	13.22
SMU_758c	*NA*	1.513	2.29
SMU_879	*msmF*	1.491	11.44
SMU_2047	*ptsG*	1.477	3.79
SMU_885	*galR*	1.472	11.09
SMU_2133c	*NA*	1.455	8.76
SMU_1960c	*NA*	1.444	7.42
SMU_1958c	*NA*	1.426	10.35
SMU_878	*msmE*	1.42	15.53
SMU_888	*galE*	1.415	11.65
SMU_1492	*lacF*	1.406	11.86
SMU_1495	*lacB*	1.405	10.33
SMU_1600	*ptcB*	1.4	5.08
SMU_314	*NA*	1.393	2.97
SMU_1956c	*NA*	1.346	4.76
SMU_434	*NA*	1.325	7.06
SMU_1493	*lacD*	1.324	11.76
SMU_1490	*lacG*	1.292	7.35
SMU_1504c	*NA*	1.273	1.61
SMU_1488c	*NA*	1.242	6.89
SMU_433	*NA*	1.223	5.32
SMU_1005	*gtfC*	1.198	5.99
SMU_120	*rpmB*	1.198	2.43
SMU_877	*agaL*	1.189	11
SMU_1505c	*NA*	1.165	3.19
SMU_889	*pbpX*	1.144	8.46
SMU_1006	*NA*	1.13	1.36
SMU_1975c	*NA*	1.129	5.44
SMU_1185	*mtlA1*	1.125	4.36
SMU_78	*NA*	1.124	9.09
SMU_409	*tsaE*	1.123	1.81
SMU_1411	*NA*	1.1	2.3

Gene ID	Genes	$\log_2$(Fold Change) (air-restricted vs. unrestricted)	$-\log_{10}$(p-value)
SMU_1548c	*NA*	1.1	2.16
SMU_1828	*NA*	1.075	7.39
SMU_1489	*lacX*	1.059	9.34
SMU_1007	*NA*	1.038	2.16
SMU_1491	*lacE*	1.028	5.51
SMU_2020	*rl16*	1.021	4.28
SMU_838	*gshR*	−1.005	8.93
SMU_248	*sufD*	−1.007	5.51
SMU_1645	*tehB*	−1.034	9.45
SMU_458	*NA*	−1.056	4.33
SMU_799c	*NA*	−1.085	3.91
SMU_383c	*NA*	−1.095	4.52
SMU_765	*ahpF*	−1.101	14.14
SMU_971	*folK*	−1.105	2.63
SMU_1128	*ciaH*	−1.11	3.4
SMU_2037	*treA*	−1.11	13.95
SMU_524	*NA*	−1.115	5.88
SMU_680	*NA*	−1.136	4.37
SMU_1048	*NA*	−1.139	1.37
SMU_1671c	*NA*	−1.141	1.94
SMU_382c	*NA*	−1.146	7.33
SMU_1419	*NA*	−1.167	2.93
SMU_403	*dinB*	−1.186	5.75
SMU_249	*nifS*	−1.189	11.19
SMU_725c	*NA*	−1.192	4.71
SMU_250	*nifU*	−1.193	7.85
SMU_478	*kguA*	−1.23	6.07
SMU_1425	*clpB*	−1.232	15.63
SMU_1034c	*xerS*	−1.247	5.67
SMU_769	*NA*	−1.264	3.89
SMU_1126	*coaA*	−1.273	4.65
SMU_2057c	*NA*	−1.294	9.05
SMU_1008	*NA*	−1.31	3.23
SMU_925	*NA*	−1.311	6.2

Gene ID	Genes	$\log_2$(Fold Change) (air-restricted vs. unrestricted)	$-\log_{10}(p\text{-value})$
SMU_1298	*rl31*	−1.422	2.33
SMU_984	*NA*	−1.447	2.12
SMU_1692	*pflA*	−1.504	6.7
SMU_260	*NA*	−1.554	7.71
SMU_1670c	*NA*	−1.56	4.81
SMU_1297	*NA*	−1.575	17.89
SMU_527	*NA*	−1.576	17.96
SMU_479	*rpoZ*	−1.586	2.52
SMU_1117	*naoX*	−1.596	17.4
SMU_132	*NA*	−1.652	19.23
SMU_764	*ahpC*	−1.678	11.1
SMU_629	*sodA*	−1.698	14.44
NA	*NA*	−1.707	5.33
SMU_1650	*end3*	−1.826	9.57
SMU_924	*tpx*	−2.01	23.69
SMU_128	*adhB*	−2.162	15.17
SMU_130	*adhD*	−2.277	23.15
SMU_929c	*NA*	−2.301	1.34
SMU_129	*adhC*	−2.357	21.02
SMU_131	*lplA*	−2.36	58.29
SMU_127	*adhA*	−2.401	29.63
SMU_1287	*NA*	−2.563	1.92
SMU_1141c	*NA*	−2.85	1.52
SMU_1988c	*NA*	−3.245	12.4
NA	*NA*	−4.302	2.35

NA: not available

Table S2. Gene list of GSEA enriched KEGG pathways (the air-restricted group vs. the air-unrestricted group).

Description	NES	−log$_{10}$(*p* adjust value)	−log$_{10}$(FDR)	Genes
Galactose metabolism	2.274	5.86	5.93	*SMU_116, SMU_886, SMU_887, SMU_1496, SMU_888, SMU_1492, SMU_1495, SMU_1493, SMU_1490, SMU_877, SMU_1491, SMU_1494;*
Phosphotransferase system (PTS)	2.271	5.34	5.41	*SMU_115, SMU_114, SMU_311, SMU_312, SMU_1596, SMU_1598, SMU_313, SMU_2047, SMU_1492, SMU_1600, SMU_1185, SMU_1491, SMU_872, SMU_980, SMU_1961c, SMU_871;*
Fructose and mannose metabolism	2.095	3.41	3.48	*SMU_115, SMU_114, SMU_311, SMU_312, SMU_308, SMU_313, SMU_1185, SMU_78, SMU_872, SMU_1182, SMU_871, SMU_1877, SMU_1183, SMU_99;*
Starch and sucrose metabolism	1.986	2.83	2.91	*SMU_1596, SMU_881, SMU_1601, SMU_1598, SMU_2047, SMU_1600, SMU_1005, SMU_1565, SMU_1564, SMU_1004, SMU_2046c;*
Citrate cycle (TCA cycle)	−1.820	2.07	2.15	*SMU_1424, SMU_128, SMU_130, SMU_129, SMU_127;*
ABC transporters	1.703	2.00	2.07	*SMU_1881c, SMU_882, SMU_431, SMU_654, SMU_880, SMU_432, SMU_879, SMU_878, SMU_1006, SMU_1007, SMU_933, SMU_1121c, SMU_1148, SMU_2119, SMU_568, SMU_1062, SMU_1079c, SMU_182, SMU_1118c, SMU_183, SMU_1519, SMU_935, SMU_1150, SMU_1078c, SMU_256, SMU_1521, SMU_1520, SMU_2149c, SMU_805c, SMU_1938c, SMU_2118, SMU_817, SMU_2117, SMU_1325, SMU_2150c, SMU_257, SMU_2116, SMU_1136, SMU_1119c,*

				SMU_242c;
Ribosome	1.652	1.44	1.51	*SMU_120, SMU_2020, SMU_2010, SMU_960, SMU_957, SMU_1626, SMU_2000, SMU_2022, SMU_2009, SMU_169, SMU_170, SMU_2021, SMU_2017, SMU_2011, SMU_1200, SMU_2002, SMU_2024c, SMU_2008, SMU_818, SMU_2014, SMU_1627, SMU_2025, SMU_2015, SMU_340, SMU_865, SMU_2007, SMU_358, SMU_846, SMU_2032;*

Table S3. Differentially expressed genes in the significantly changed KEGG pathways.

Gene ID	Gene Name	KEGG pathway
SMU_116	*lacD*	Galactose metabolism
SMU_877	*agaL*	
SMU_886	*galK*	
SMU_887	*galT*	
SMU_888	*galE*	
SMU_1490	*lacG*	
SMU_1491	*lacE*	
SMU_1492	*lacF*	
SMU_1493	*lacD*	
SMU_1495	*lacB*	
SMU_1496	*lacA*	
SMU_78	*fruA*	Fructose and mannose metabolism
SMU_114	*NA*	
SMU_115	*NA*	
SMU_311	*NA*	
SMU_312	*NA*	
SMU_313	*NA*	
SMU_308	*NA*	
SMU_1185	*mtlA1*	
SMU_881	*gtfA*	Starch and sucrose metabolism
SMU_1005	*gtfC*	
SMU_1596	*ptcC*	
SMU_1598	*ptcA*	
SMU_1600	*ptcB*	
SMU_1601	*bgl*	
SMU_2047	*ptsG*	
SMU_114	*NA*	Phosphotransferase system (PTS)
SMU_115	*NA*	
SMU_311	*NA*	
SMU_312	*NA*	
SMU_313	*NA*	
SMU_1185	*mtlA1*	
SMU_1491	*lacE*	

Gene ID	Gene Name	KEGG pathway
SMU_1492	*lacF*	
SMU_1596	*ptcC*	
SMU_1598	*ptcA*	
SMU_1600	*ptcB*	
SMU_2047	*ptsG*	
SMU_431	*NA*	ABC transporters
SMU_432	*NA*	
SMU_654	*pepB*	
SMU_878	*msmE*	
SMU_879	*msmF*	
SMU_880	*msmG*	
SMU_882	*msmK*	
SMU_1006	*NA*	
SMU_1007	*NA*	
SMU_1881c	*NA*	
SMU_127	*adhA*	Citrate cycle
SMU_128	*adhB*	
SMU_129	*adhC*	
SMU_130	*adhD*	

NA: not available

Table S4. List of Primers

**Primer Name**	**Sequence 5' –> 3'**
16SrRNA_F	ACCAGAAAGGGACGGCTAAC
16SrRNA_R	TAGCCTTTTACTCCAGACTTTCCTG
dpr_F	ACCAAGGCTGTACTTAATCAGG
dpr_R	AGGATGCAGATAAAGGAAGCC
galK_F	TGGGAGCTGAAAAGAAGGCT
galK_R	GTCACCAAGGTCAAGCGGTA
galT_F	ATTCGTCTGGCTTGGCGTAA
galT_R	CCATCGCGTTTTCGAGCAAT
galE_F	CCTGCAGAAAAAGCAGCTCG
galE_R	CAAATTGCGGTTTCCAGCCA

**Primer Name**	**Sequence 5' –> 3'**
gftD_F	ACAGCAGACAGCAGCCAAGA
gftD_R	ACTGGGTTTGCTGCGTTTG
gtfA_F	ATAGCTGCTGTATCGGGTGC
gtfA_R	ACGGTCAACCTTGCTCGAAT
gtfB_F	AGCAATGCAGCCAATCTACAAAT
gtfB_R	ACGAACTTTGCCGTTATTGTCA
gtfC_F	TGGCAATGATGGCTATGCCT
gtfC_R	CTGCCACGACCAAAGTCTCT
gyrA_F	CCAAGAATCTGCTGTCCG
gyrA_R	TTGCGACTATCGCTATGTG
lacE_F	AACTTGCGGAGCCAAGATGA
lacE_R	AGCCGCAGAAGAATACGGAG
lacF_F	GCTACAAGCTCTTCTGCCCT
lacF_R	AGCCTATGCAGGAGATGCAC
lacG_F	ACGGCTCCATACCCTTTTGG
lacG_R	TGAACCTGCCAGCGATTTCT
msmE_F	CCGTTTGCTTTAGCGGGAAC
msmE_R	TGGTTGGGAAAAGCGAAGGT
msmF_R	GGTCAGTCGAGTTGCCCTAC
msmF_R	CGCCACCTGTTAAGGCAAAG
msmG_F	GGCCTGGCTAATATGTGGGG
msmG_R	GAGTTGTTGCGTGCATTGGT
msmK_F	AGTTGCGGGTATCTATGCGG
msmK_R	GCTAAGGTCATGGCTTCCGT
nox_F	GGAATCGTTAGAAGCAAAAGGTG
nox_R	TGACCAAAGCAGTAACCCTC
SMU_113_F	GGGATAGCTGCCCCTTTTGT
SMU_113_R	TAGCCTGCATTGAGCAGCTT
SMU_115_F	GCTTTGCCATTCCTCATGCC
SMU_115_R	ATGCCATTCAACACCCGTCT
lacA_F	CGCATTATTATGGCCGCGTG
lacA_R	TATGGTGCGGGCAGCTTTAT
lacB_F	TAATAGCGCCGCCAAAGCTA
lacB_R	CCGGGTGTTAGAACGGCATT
lacC_F	*CCGTTGAATCCCCTGAACCA*

**Primer Name**	**Sequence 5' –> 3'**
lacC_R	*GGTTCTCAAGGTGCTTTTGCC*
lacD2_F	ACAAGCGCGTGATAGGGAAG
lacD2_R	CGACTGGTTGTGTTCGCATC
lacD_F	TGGGCCATCACCAAAACCTT
lacD_R	CCATTACTGATGCCGCAAGC
SMU_308_F	TCCTCATGGGCAATACGAGC
SMU_308_R	TTGGTTCCCGCATAAGCACT
SMU_311_F	TGGCGATTAGTATGGGACGC
SMU_311_R	CCTGATGCAATCCCCATCCA
SMU_312_F	ATTCAAGGCTCAGGTGTGGG
